# Enhanced Hardness and Tribological Properties of Copper-Based Steel Backing Self-Lubricating Materials with Y_2_O_3_ Micro-Doping

**DOI:** 10.3390/ma18030560

**Published:** 2025-01-26

**Authors:** Mingmao Li, Ningkang Yin, Zhaokui Jei, Zhiying Liu, Jinhan Zhang, Hao Zeng, Hao Huang, Jingxuan Liu

**Affiliations:** 1 Faculty of Materials Metallurgy and Chemistry, Jiangxi University of Science and Technology, Ganzhou 341000, Chinayningk@163.com (N.Y.);; 2 Jiangxi Hongda Self Lubricating Bearing Co., Ltd., Yingtan 335425, China; 3 Yingtan Inspection, Testing and Certification Institute, Yingtan 335001, China

**Keywords:** copper-based self-lubricating material, Y_2_O_3_-Cu based powder metallurgy, friction and wear, dispersion strengthening, fretting fatigue

## Abstract

The copper-based steel backing material is prepared using a combination of mechanical alloying and secondary sintering methods. The effect of Y_2_O_3_ content on the microstructure, hardness, and tribological properties of the copper-based self-lubricating layer is investigated. The results demonstrate that the addition of Y_2_O_3_ enhances the strength of the copper-based self-lubricating layer. Graphite and Y_2_O_3_ act synergistically to form a three-dimensional supporting framework, thereby boosting the overall strength of the copper-based composite material and increasing its Brinell hardness by 27%. Additionally, the incorporation of Y_2_O_3_ effectively improves the tribological properties of the composite material, significantly reducing wear during the friction process and decreasing the wear rate by 77%. Under the experimental conditions, the optimal Y_2_O_3_ content is determined to be 1 wt%.

## 1. Introduction

Metal matrix self-lubricating composites are a class of advanced materials created by incorporating one or more lubricants into the matrix material, which may consist of metals, alloys, or intermetallic compounds. These composites have garnered significant attention from researchers owing to their exceptional lubrication performance during operation [1,2]. Copper-based steel backing (CBSB) self-lubricating materials represent a unique category of dual-layer metal composites. They are formed by integrating a copper-based self-lubricating layer with the working surface of steel. This innovative material not only preserves the outstanding self-lubricating properties but also capitalizes on the thermal expansion coefficient compatibility and robust load-bearing capacity of steel. As a result, it is well-suited to fulfill the stringent operating conditions of various complex large-scale equipment, holding great promise for applications in fields such as machinery, automotive braking systems, and bearings.

Copper-based powder metallurgy self-lubricating friction materials have found widespread application across diverse industries. Copper’s remarkable ductility, combined with its superior electrical and thermal conductivity, has facilitated its extensive utilization in sectors like electronic communication [3,4]. A study by Yang Song et al. [5] meticulously investigated the impact of carbon fiber powder on copper-based self-lubricating composite materials. The research outcomes indicated that the addition of 0.2 wt% carbon fiber powder led to a modest improvement in the materials’ friction and wear characteristics. Nevertheless, the hardness of the resultant material was a relatively low 17 HBW. This finding suggests that while carbon fiber can substantially enhance the friction performance of pure copper, its capacity to elevate the material’s mechanical properties is somewhat limited, as reflected in the consistently low hardness measurement. Hao Li [6] and his research team utilized Spark Plasma Sintering (SPS) to fabricate copper-based self-lubricating composites, incorporating nickel-plated graphite and WS_2_ into the matrix. This innovative approach led to notable improvements in the material’s strength and wear resistance. Despite these advancements, the SPS technique is hampered by its high cost and scalability challenges, particularly in the context of large-scale industrial applications. As a result, the research community has increasingly turned its attention towards iron-based self-lubricating materials. These materials boast high mechanical strength and offer a cost-effective solution. Regrettably, when these iron-based materials are subjected to friction against counterparts like cast iron or steel, they exhibit significant fluctuations in the friction coefficient and demonstrate a weak anti-wear effect, thereby struggling to accommodate the diverse demands of various operating conditions. A wealth of studies has highlighted the synergistic benefits of combining copper and iron as the matrix for friction materials. Such composites not only harness the excellent mechanical properties and thermal conductivity of both metals but also deliver enhanced friction performance and cost-effectiveness. In a notable contribution to this field, FU Chuanqi [7] and colleagues augmented copper-iron-based self-lubricating friction materials with 0.5 wt% graphene. This modification yielded a remarkable 12.39% increase in hardness, although the material’s overall strength still fell short of optimal levels.

In the realm of mechanical manufacturing, the relentless pursuit of precision and efficiency has propelled copper-based steel-backed self-lubricating materials to the forefront as a promising new class of friction materials [8]. The quest for copper-based steel-backed self-lubricating materials that exhibit high strength, wear resistance, and low friction coefficients has emerged as a focal point of research endeavors. To satisfy the spectrum of operational requirements, this study proposes a novel approach by employing metallic copper as the primary lubricating matrix and metallic iron as the auxiliary bearing material, thereby engineering advanced copper-based composite materials. The resultant composites seamlessly merge the mechanical attributes and thermal conductivity of copper and iron, while also boasting commendable friction performance and cost advantages [9]. Moreover, the integration of solid lubricant graphite powder into the copper-based composites serves to markedly amplify their self-lubricating capabilities. When these composite materials are sintered onto steel substrates, the resulting copper-based steel-backed self-lubricating bearings exhibit an unparalleled level of overall performance [10].

Addressing the wettability challenges inherent in the interaction between dissimilar metals and non-metals is pivotal to further optimizing the comprehensive performance of these materials. This study innovatively introduces second-phase particles, characterized by high thermal stability and a uniform dispersion, into the copper matrix. The strategic incorporation of these particles is designed to hinder dislocation movement, consequently fortifying the strength of the copper matrix. The judicious selection of appropriate oxide fine particles is thus of paramount importance. Among the array of common oxide particles, Y_2_O_3_ stands out due to its elevated formation enthalpy compared to Al_2_O_3_ and Cr_2_O_3_ at 1300 K. The relatively low solubility of Y in the copper matrix further underscores Y_2_O_3_’s enhanced stability at elevated temperatures. Additionally, the Y element plays a crucial role in reacting with impurities such as sulfur, calcium, and oxygen at the interface, leading to the formation of refractory compounds that effectively purify the copper matrix interface [11,12,13,14,15,16,17].

The primary objective of the present study is to pioneer the development of copper-based self-lubricating materials reinforced with Y_2_O_3_ particles. By meticulously adjusting the Y_2_O_3_ content, we aim to strike an optimal balance between the material’s strength and its friction-wear performance. To achieve this, we will leverage a suite of testing techniques and experimental methodologies, integrating macroscopic and microscopic analyses to delve into the intricate microstructure and properties of these materials, thereby unlocking their full potential in practical applications.

## 2. Materials and Methods

### 2.1. Sample Preparation

**Figure 1:**
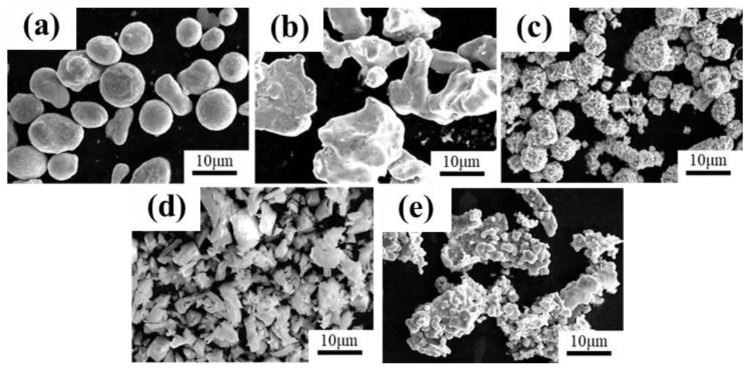
SEM images of the raw powder materials morphology: (**a**) Cu powder; (**b**) Fe powder; (**c**) Ni powder; (**d**) Y_2_O_3_ powder; (**e**) Graphite powder.

Figure 1 illustrates the SEM photographs of the microscopic morphology of the experimental raw material powders. Four types of powder materials were prepared using mechanical alloying for the experiments, and the sample numbers and mass compositional are shown in Table 1. The purity levels of the Cu powders (Nangong Zhaohuan Alloy Welding Materials Co., Ltd., Nangong, China), Fe powders (Nangong Chunxu Metal Materials Co., Ltd., Nangong, China), Ni powders (Hunan Jinkun New Materials Co., Ltd., Zhuzhou, China), Graphite powders (Shenzhen Jiasheng New Materials Co., Ltd., Shenzhen, China), and Y_2_O_3_ powders (Shanghai Lingyao New Materials Co., Ltd., Shanghai, China) powders were all above 99.9%, which serve as the base materials for the self-lubricating composite layer. The particle size of the composite powder is between 40 and 50 μm. In addition, Y_2_O_3_ particles (average particle size of 10 μm) were selected as the reinforcing particles to be doped into the mixed powder. The four powders are displayed in Table 1. The mixed powders with different additive contents were abbreviated as CFN0-Y, CFN0.5-Y, CFN1.0-Y, and CFN1.5-Y, respectively.

**Figure 2:**
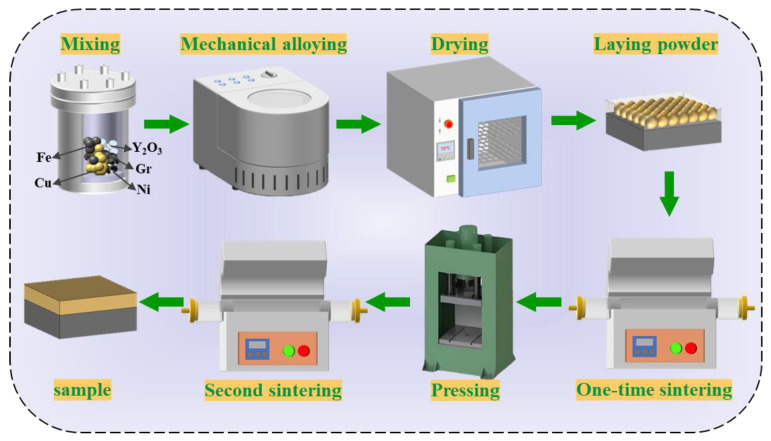
The schematic diagram of the CBSB materials preparation process.

The schematic diagram of the CBSB materials preparation process is illustrated in Figure 2. The base powders and Y_2_O_3_ powders were mixed via a ball mill (QM-3SP4, CN, Nanjing NANDA Instrument Co., Ltd., Nanjing, China) with ethanol used as the milling medium. While mixing, the ratio of ball milling balls to powder was 5:1, the ball milling balls are tungsten steel balls with two sizes of Φ = 10 mm and 5 mm, and the rotating speed is 300 r/min, and the ball milling time is 6 h to ensure uniform distribution of Y_2_O_3_ within the matrix. After milling was completed, the composite powders were vacuum-dried.

The dried composite powders were evenly spread on a 45# steel plate, leveled by vibration, and then subjected to the first high-temperature sintering. The sintering is conducted in a vacuum reduction tubular furnace (TST1700-80, CN, Shanghai TESE electric furnace equipment Technology Co., Ltd., Shanghai, China) with hydrogen protection, and a sintering temperature is 950 °C, a heating rate of 10 °C/min, and a sintering duration of 3 h. After completion, the samples were cooled in the furnace. Following the initial sintering, the samples were placed in a hydraulic press for compaction with a deformation amount of 20%. Subsequently, the secondary high-temperature sintering began, with the sintering temperature rising to 1050 °C, while other parameters remained consistent with the first sintering. Finally, the process was completed and CBSB materials were obtained.

### 2.2. Sample Characterization

The microstructure of the grounded powders and CBSB sample were visualized with the TESCAN MIRA field emission gun scanning electron microscope (S8000, CZ, TESCAN, Brno, Czechia) combined with the Oxford X-act energy dispersive spectroscopy (Ultim Extreme, Oxford Instruments, Abingdon, UK). The composition of phases within the customized composites was analyzed using X-ray diffraction (D8 ENDEAVOR, Bruker, Billerica, MA, USA). The density of the copper-based composite material was measured using the Archimedes’ water displacement method. The relative density is the value obtained by dividing the real density by the theory, which reflects the degree of densification of the alloy. It should be noted that all the samples used for characterization and subsequent testing were cut from the copper-based self-lubricating layer using an electric discharge wire cutting machine (X8, Nanchang Guosai NC Electromechanical Equipment Co., Ltd., Nanchang, China).

### 2.3. Mechanical Testing

The indentation area of the Brinell hardness test is relatively large, which can reflect the average performance of a large area while avoiding significant measurement errors caused by pores. In the context of this research, Standard GB/T 231.1-2018 [18] was adopted for the hardness test of the copper layer. The Brinell hardness tester (HBS-3000M, CN, Shanghai Lidun Instrument Testing Co., Ltd., Shanghai, China) was used to test the hardness of self-lubricating composite materials. Hardened steel balls with a ball diameter of 10 mm were pressed into the specimen surfaces with a test force of 1000 kg. The holding time was 15 s, the impression diameter was measured, and the Brinell hardness was calculated at this point. Each specimen was measured at 5 different positions, and the average value was recorded.

### 2.4. Tribological Testing

**Figure 3:**
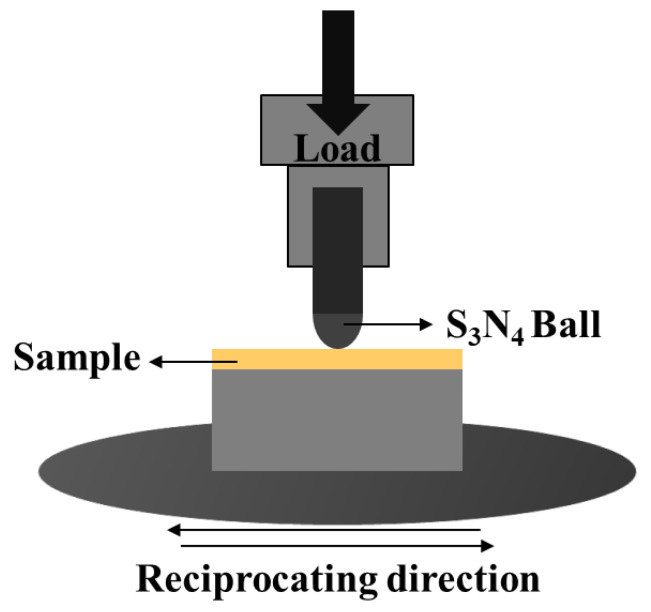
Schematic diagram of reciprocating friction test device.

The friction coefficient of the materials was measured using a UMT friction and wear testing machine (UMT-3, Mouser Electronics, Mansfield, TX, USA) with a 6 mm diameter Si_3_N_4_ ball as the counter material. The tests (ASTM G 133-22 [19]) were conducted under a load pressure of 30 N with a linear reciprocating motion of 10 mm for 30 min at a frequency of 5 Hz. All samples underwent vacuum oil absorption and surface polishing treatments. A schematic diagram of the testing apparatus is shown in Figure 3. The wear volume was measured using a weighing method, where a precision balance was employed to weigh the samples before and after the friction tests. The three-dimensional morphology of the worn surface was captured using a white light interferometric three-dimensional profilometer (Retc instruments, San Jose, CA, USA).

## 3. Results and Discussion

### 3.1. Morphology of Mechanically Alloyed Particles

**Figure 4:**
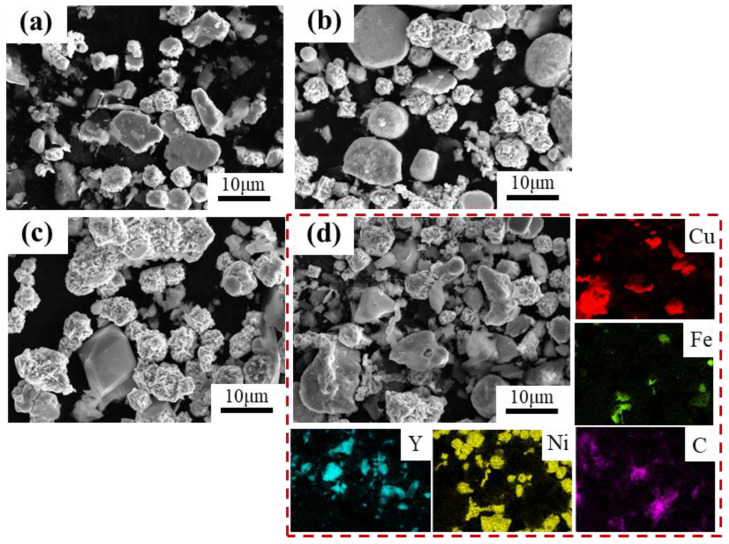
SEM images of the powder obtained by ball milling: (**a**) CFN0-Y; (**b**) CFN0.5-Y; (**c**) CFN1.0-Y; (**d**) SEM and corresponding EDS analysis of CFN-1.5Y.

Figure 4 shows the SEM and EDS images of the mixed powders with different Y_2_O_3_ addition ratios (0 wt.%, 0.5 wt.%, 1 wt.%, and 1.5 wt%) after 6 h ball milling. In Figure 4a–d, it can be observed that the powders have fragmented during ball milling, resulting in the formation of irregular shapes for most particles, while some particles have gradually flattened. The calculated average particle size is 15 μm. Additionally, it can be observed that as the amount of Y_2_O_3_ added increases, the number of irregularly shaped particles first increases and then decreases. However, as a hard particle, Y_2_O_3_ has no significant change in shape and size during mechanical alloying process. As seen in Figure 4d, uniform dispersion of Cu powder, Ni powder, Fe powder, and graphite powder has been achieved. The fine Y_2_O_3_ particles do not agglomerate but are dispersed throughout the mixed powder, which is beneficial for the sintering and densification of the composite powder [20].

### 3.2. Microstructure Characteristics of Sintered Materials

**Figure 5:**
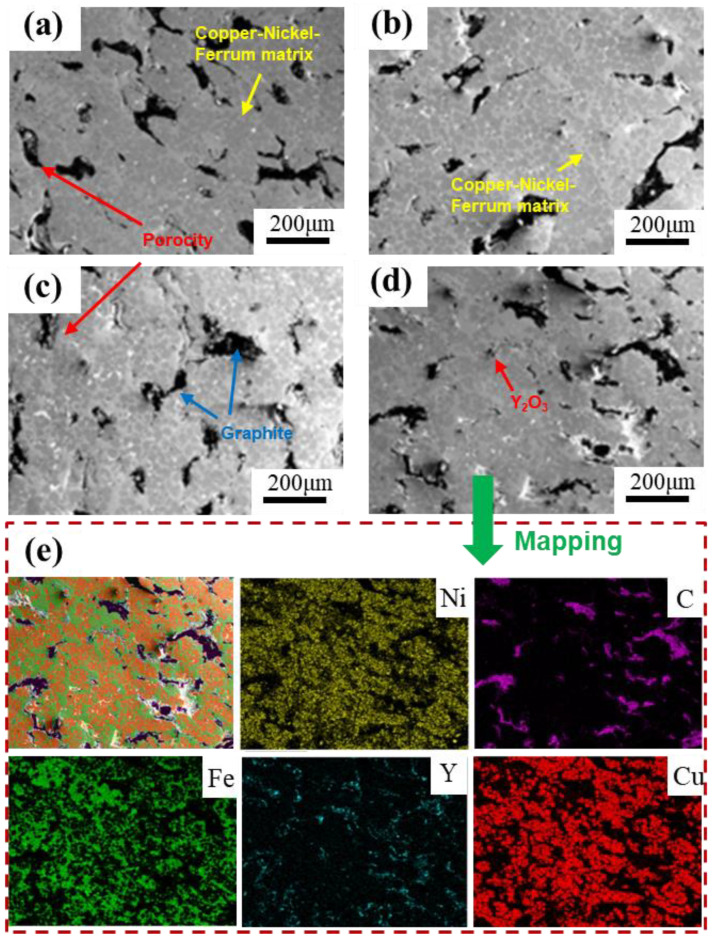
SEM and EDS images of the sample surface: (**a**) CFN0-Y; (**b**) CFN0.5-Y; (**c**) CFN1.0-Y; (**d**) CFN1.5-Y; (**e**) corresponding EDS analysis of (**d**).

Figure 5 shows the surface morphology of the sintered samples with different Y_2_O_3_ addition ratios. As shown in Figure 5a–d, pores of varying sizes were observed on the surfaces of the four kinds of samples. As the Y_2_O_3_ content increases, the number of pores on the sample surfaces shows an increasing trend. The Y_2_O_3_ particle is a kind of hard ceramic particle, which exhibits poor wettability with Cu [21,22]. This characteristic would hinder the growth of the sintering neck, as well as the spheroidization and shrinkage of closed pores during the sintering process, ultimately resulting in a decrease in the density of the sample. This phenomenon was further exacerbated with the increase in Y_2_O_3_ content.

EDS analysis was conducted on the CFN1.5-Y sample. As shown Figure 5e, the C element is primarily enriched around the pores. This is due to the use of copper-coated graphite in this experiment, which improves the wettability between graphite and the metal matrix [23], allowing graphite to adhere closely to the inner walls of the pores. Moreover, a large number of yttrium oxide particles are evenly distributed within the pores, working in conjunction with the graphite to form a three-dimensional supporting structure throughout the material. In addition, due to the excellent plasticity of graphite [24], the presence of this structure can absorb some external stress when subjected to applied stress, helping to maintain structural stability, and thereby enhancing the overall mechanical strength of the material. The hardness values measured for each sample will be discussed in the following sections.

**Figure 6:**
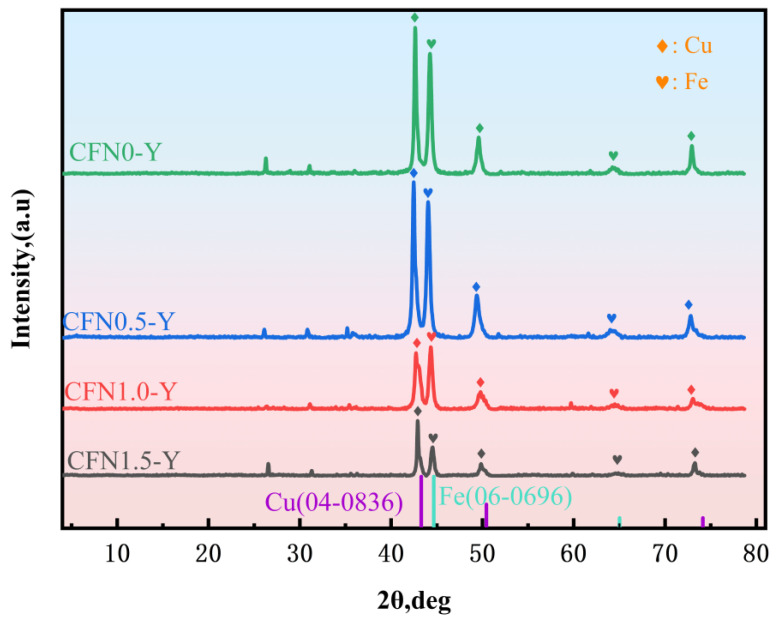
XRD patterns of samples with different Y_2_O_3_ contents.

Figure 6 illustrates the XRD patterns of sintered samples with varying Y_2_O_3_ contents. No impurity characteristic peaks were detected, aside from those corresponding to Cu, Fe, and C in the diffraction patterns. At a sintering temperature of 950 °C, it is inevitable that some diffusion of elements will occur. However, no metal bond compounds have been formed. According to the Cu-Fe alloy phase diagram, the copper and iron powders in the self-lubricating layer do not form a solid solution. Owing to the low concentration of Y_2_O_3_ in the composite materials, its characteristic peaks are not distinctly visible. However, in comparison to the sample devoid of Y_2_O_3_, the primary diffraction peak of Cu in the Y_2_O_3_-containing samples exhibits a slight shift towards a lower angle. In accordance with Bragg’s equation, a decrease in angle signifies an increase in lattice constant and interplanar spacing, indicating that the crystal structure has undergone doping [15]. This observation implies that Y_2_O_3_ not only resides in closed pores but also partially reacts with the Cu matrix. Consequently, the copper matrix lattice experiences distortion, attributed to the synergistic effects of plastic deformation and the incorporation of Y_2_O_3_ particles.

### 3.3. Hardness and Density

**Figure 7:**
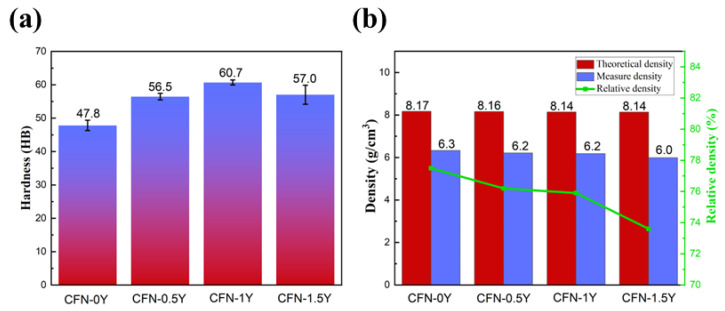
Average hardness and actual density of the sample: (**a**) Average hardness; (**b**) Density.

In the Brinell hardness tests, five testing points were conducted in the same direction after polishing the surfaces of the four composite materials, and then their average values were calculated. Figure 7a shows the average hardness of each sample. The results indicate that the addition of Y_2_O_3_ significantly improved the hardness of the sample. The composite material exhibits the highest hardness value of HB60.7 when the addition of Y_2_O_3_ is 1 wt.%, and the hardness increased by 15.5% compared to the sample without Y_2_O_3_ added. The added Y_2_O_3_ material is a hard ceramic, which acts as a second-phase hard ceramic particle and typically enhances the mechanical properties of the material, providing structural support and reducing the plastic deformation of the matrix under pressure. During the sintering process of the material, an appropriate amount of yttrium oxide can reduce the flowability of the copper matrix and enhance the bonding between graphite and the copper matrix. Therefore, the surface hardness of the samples was significantly increased. This result is attributed not only to the strengthening effect of Y_2_O_3_, but also further demonstrates that the good dispersion of fine particles in the Cu matrix stabilizes the ceramic particles around the grain boundaries. These particles act as effective barriers, impeding dislocation movement within the matrix and enhancing the resistance to indentation. In addition, the interfacial reaction zone between graphite and Y_2_O_3_ can effectively transfer stress, and through the load transfer mechanism, distribute the primary load to the matrix, thereby enhancing the load-bearing capacity and hardness of the self-lubricating material [25]. It is worth noting that as the amount of Y_2_O_3_ added increases, the porosity of the composites further enlarges, leading to a reduction in the density. Sample densities and relative densities of the samples are shown in Figure 7b. When the impact of reduced density outweighs the reinforcing effect of the second-phase hard particles, the hardness of the composite material will decrease. This explains why the hardness of the CFN1.5-Y sample is lower than that of the CFN1.0-Y sample showed in Figure 7a.

The density of powder metallurgy materials is an important factor affecting their mechanical properties. The relative density (*ρ_rd_*) is the ratio of the actual measured density (*ρ_ad_*) to the theoretical density (*ρ_td_*) expressed as a percentage [25], which is given by the formula:

(1) $$\rho_{rd}=\frac{\rho_{ad}}{\rho_{td}}\times 100\%$$

The theoretical density calculation formula for materials is:

(2) $$\rho_{td}=\rho_{1}w_{1}+\rho_{1}w_{1}+\cdots +\rho_{i}w_{i}$$

where *ρ_i_* is the density of the *i*-th component added, in g/cm^3^; and *w_i_* is the mass fraction of the *i*-th component added, in wt.%.

### 3.4. Analysis of Tribological Properties

Friction and wear tests were conducted three times for each sample to ensure the reliability and repeatability of the test results. The factors such as material hardness, normal force, and sliding distance have a significant impact on the wear rate. The Archard wear model is used to calculate the wear rate of the material [26]. The formula is shown as follows:

(3) $$K=\frac{VH_{Pa}}{SF}$$

The wear volume of the material is calculated using the mass loss method:

(4) $$V=\frac{∆m}{\rho_{ad}}$$

The conversion formula between Brinell Hardness (HB) and material hardness (Pa) is as follows:

(5) $$H_{Pa}=\frac{1000\times HB}{D^{2}}$$

where *V* is the wear volume, m^3^; *S* is the sliding distance, m; *K* is the Archard wear rate, m^3^/N·m; *F* is the normal force, N; *H* is the material hardness, Pa; *Δm* is the mass loss due to wear, g; and *D* is the diameter of the indenter, mm.

Table 2 provides a detailed list of the average coefficient of friction and wear rate for each sample. The research findings indicate that the average coefficient of friction for samples with varying Y_2_O_3_ content does not show significant differences compared to the samples without Y_2_O_3_. However, it is particularly noteworthy that the samples with added Y_2_O_3_ exhibit a significantly reduced wear rate. Among these, the sample containing 1 wt.% Y_2_O_3_ performs the best, with an average coefficient of friction of only 0.0993 and the lowest wear rate of 0.545 m^3^/N·m.

**Figure 8:**
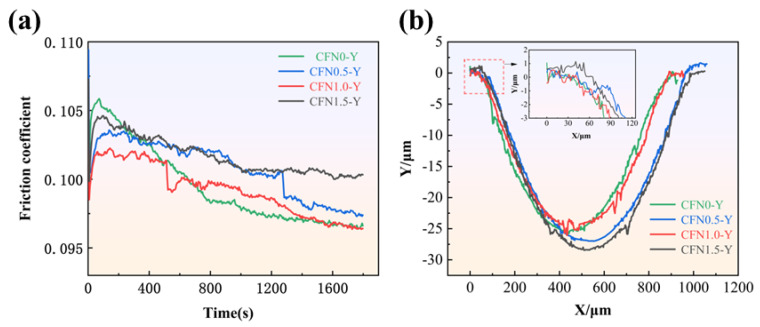
(**a**) Relationship between friction coefficient and time; (**b**) Grinding scar cross-sectional profile.

To further elucidate the friction process, the friction coefficient versus time graph shown in Figure 8a was analyzed. It is observed that the friction coefficient fluctuates over time. During the initial minutes of testing, the friction coefficients of all four samples exhibited significant variation due to direct contact between the sample and the friction pair, with the load already set to the desired value. At this stage, the copper-based metallic material primarily provided a lubricating effect, resulting in a generally high friction coefficient. As the friction progressed, despite the load remaining constant, rapid frictional work generated substantial heat, leading to an increase in sample temperature. This caused the lubricating oil stored in the pores to expand and be released to the friction interface, quickly forming a lubrication film on the surface, which significantly reduced the friction coefficient. Notably, the CFN1.0-Y sample experienced the most pronounced decrease in friction coefficient, attributed to the excellent high-temperature stability of Y_2_O_3_ and its effect on material density [27]. Over prolonged friction, the contact surface expanded and began to deteriorate, resulting in plastic deformation and adhesive wear on the material surface. Graphite stored in the pores began to diffuse to the surface under pressure, exerting lubrication effects. Subsequently, the range of fluctuation in the friction coefficient decreases and stabilizes within a certain range.

**Figure 9:**
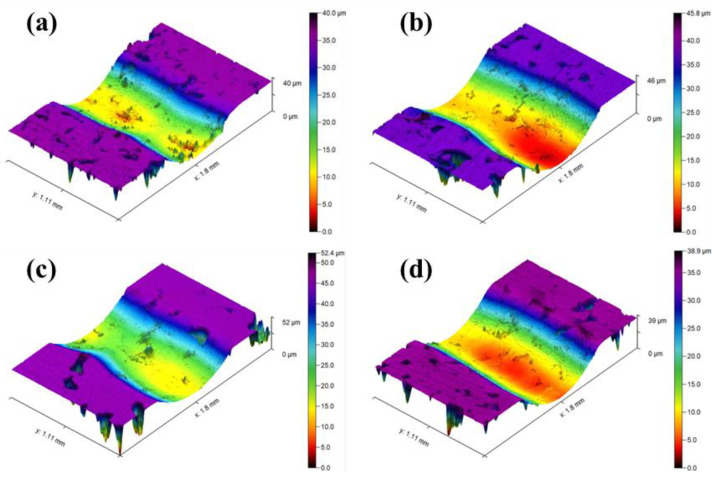
Three dimensional contour diagram of sample abrasion: (**a**) CFN-0Y; (**b**) CFN-0.5Y; (**c**) CFN-1.0Y; (**d**) CFN-1.5Y.

Figure 8b shows the cross-sectional profile of the wear track. The results indicate that all four samples exhibit good wear resistance. The maximum wear track depth for all four samples is less than 29 μm, with the CFN1.0-Y sample showing the smallest wear track depth of only 26.992 μm, indicating that this sample has the strongest resistance to plastic deformation and the highest wear resistance. It is noteworthy that all four sets of specimens exhibit portions elevated above the reference plane, with CFN-1.0Y showing the lowest elevation. This further suggests that CFN-1.0Y demonstrates superior anti-wear performance, while the other three sets of specimens display inferior wear resistance. As a result, the width and depth of the groove are larger, and the raised area on both sides is more obvious, which means that the amount of wear is increased. The addition of Y_2_O_3_ enhances the material’s yield strength and hardness (as shown in Figure 6), thereby improving wear resistance. Additionally, from the three-dimensional profile in Figure 9, it can be observed that the wear track forms an elliptical pit, with the maximum depth at the center of the track. This suggests that the contact stress is most concentrated in the center region of the wear track, leading to the most severe plastic deformation. Therefore, a smaller wear track depth indicates less wear, further confirming that the CFN1.0-Y sample has superior wear performance.

**Figure 10:**
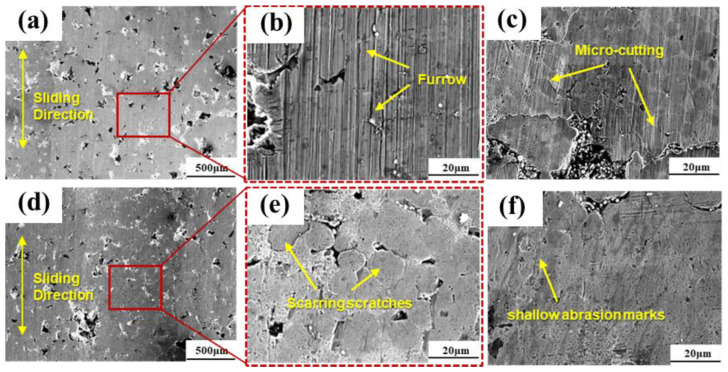
Scanning image of sample abrasion marks: (**a**–**c**) CFN-0Y, (**d**–**f**) CFN-1.0Y and (**c**,**f**) Surface morphology during the initial stage of the test.

The scanning images of each sample after the friction and wear tests are shown in Figure 10. From the wear surfaces, it can be observed that the CFN0-Y sample, which does not contain Y_2_O_3_, exhibits a significant number of “grooves” compared to the samples with added Y_2_O_3_. This indicates that the wear mechanism for the CFN0-Y sample is predominantly abrasive wear. Due to its lower porosity, the release of lubricating oil and graphite to form a lubricating film is delayed during the initial stages of friction. As a result, wear particles generated by the normal loads on the sample surface become embedded into the friction surface. The friction force during sliding, influenced by the “grooving effect,” creates grooves on the sample surface, forming the observed “plow” wear tracks [28,29,30], as shown in Figure 10c,f. As friction continues, the lubricating graphite stored in the pores is extruded to form a lubricating film, at which point the wear mechanism shifts to adhesive wear. However, the presence of multiple parallel grooves on the friction surface exacerbates the detachment and destruction of the graphite lubricating film, generating more layered or flaky graphite debris, which enhances the lubricating effect of the graphite and effectively controls wear. For the CFN1.0-Y sample, the excellent wear resistance and high-temperature stability of Y_2_O_3_ allow the lubricating oil stored in the pores to quickly release and form a lubricating film [31], resulting in a swift transition from abrasive wear in the initial friction stages to slight adhesive wear. On the other hand, the CFN0.5-Y and CFN1.5-Y samples, which have lower hardness, exhibit larger wear track cross-sections and deeper wear tracks, as shown in Figure 10b,e. Due to the fact that adhesive wear volume is directly proportional to the normal load and sliding distance, and inversely proportional to the material hardness [25], the addition of an appropriate amount of Y_2_O_3_ increases the hardness of the CFN 1.0-Y sample to the highest level, thereby reducing the degree of surface wear. In this case, the adhesive points break along the interface, forming a relatively smooth wear surface with only minor scratches, making it difficult for significant plastic deformation and adhesive transfer to occur, thus reducing the wear volume. In contrast, the CFN0-Y sample, which has a slightly lower hardness, may experience shear fracture within the material, leading to severe material flow and transfer, resulting in a larger wear volume.

## 4. Conclusions

This study employs mechanical alloying combined with vacuum secondary sintering and pressing processes to investigate the microstructure, mechanical properties, and friction-wear performance of Y_2_O_3_-reinforced copper-based self-lubricating composites with Y_2_O_3_ powder contents of 0 wt.%, 0.5 wt.%, 1.0 wt.%, and 1.5 wt.%. The main findings and conclusions are as follows:

(1) The microstructure of the mixed composite powders and sintered samples was analyzed by SEM and XRD. The doping of Y_2_O_3_ will increase the porosity of the composite and deteriorate with the increase of the addition amount. However, Y_2_O_3_ particles are evenly distributed in the pores and working in conjunction with graphite to form a three-dimensional support structure for the material as a whole.
(2) The addition of Y_2_O_3_ can significantly improve the hardness of the composite. The CFN-1Y exhibited the highest hardness of around HB60.7, which is 15.5% higher than that of CFN-0Y. The relative density of the composites decreased with increasing Y_2_O_3_ content. For instance, the relative density of CFN-0Y is 77.5%, while the samples with Y_2_O_3_ contents of 0.5 wt.%, 1 wt.%, and 1.5 wt.% have relative densities of 76.2%, 75.9%, and 73.6%, respectively.
(3) The experimental sample with 1 wt.% Y_2_O_3_ content exhibited the lowest average friction coefficient and decreased the wear rate by 77%. The regulation of porosity by Y_2_O_3_ allows for effective and timely release of the lubricant, which protects the material and reduces wear. This beneficial effect contributes to the improved tribological performance of the composite.

## Figures and Tables

**Table 1:** Nominal composition of materials (wt.%).

Sample	Cu	Fe	Ni	Graphite	Y_2_O_3_
CFN0-Y	41.00%	REM	15.00%	6.00%	0.00%
CFN0.5-Y	41.00%	REM	15.00%	6.00%	0.50%
CFN1.0-Y	41.00%	REM	15.00%	6.00%	1.00%
CFN1.5-Y	41.00%	REM	15.00%	6.00%	1.50%

**Table 2:** Average Coefficient of Friction and Wear Rate for Each Sample.

	CFN0-Y	CFN 0.5-Y	CFN 1.0-Y	CFN 1.5-Y
Avg. COF	0.0995	0.1008	0.0993	0.1017
Avg. K (m^3^/N·m)	2.378 × 10^−11^	1.346 × 10^−11^	0.545 × 10^−11^	0.705 × 10^−11^

## Data Availability

The original contributions presented in this study are included in the article. Further inquiries can be directed to the corresponding author.
